# Preventing Complications from High-Dose Rate Brachytherapy when Treating Mobile Tongue Cancer via the Application of a Modular Lead-Lined Spacer

**DOI:** 10.1371/journal.pone.0154226

**Published:** 2016-04-29

**Authors:** Shumei Murakami, Rinus G. Verdonschot, Naoya Kakimoto, Iori Sumida, Masateru Fujiwara, Kazuhiko Ogawa, Souhei Furukawa

**Affiliations:** 1 Department of Oral and Maxillofacial Radiology, Osaka University Graduate School of Dentistry, Suita, Osaka, Japan; 2 Department of Radiation Oncology, Osaka University, Graduate School of Medicine, Suita, Osaka, Japan; North Shore Long Island Jewish Health System, UNITED STATES

## Abstract

**Purpose:**

To point out the advantages and drawbacks of high-dose rate brachytherapy in the treatment of mobile tongue cancer and indicate the clinical importance of modular lead-lined spacers when applying this technique to patients.

**Methods:**

First, all basic steps to construct the modular spacer are shown. Second, we simulate and evaluate the dose rate reduction for a wide range of spacer configurations.

**Results:**

With increasing distance to the source absorbed doses dropped considerably. Significantly more shielding was obtained when lead was added to the spacer and this effect was most pronounced on shorter (i.e. more clinically relevant) distances to the source.

**Conclusions:**

The modular spacer represents an important addition to the planning and treatment stages of mobile tongue cancer using HDR-ISBT.

## Introduction

According to a report by the World Health Organization (WHO) there is a marked rise in the incidence rates for oral cancers in various countries and regions [1] and it has been projected that in the US alone between 2010 and 2020 yearly about 3.6 to 4.4 billion dollar will be spent on the treatment of head and neck cancer [2]. Within the head and neck regions, carcinoma of the tongue is one of the most common types of oral cancer [3]. When treating this particular type of cancer interstitial brachytherapy (ISBT) is an important treatment option as it may greatly preserve tongue function and mobility (compared to other treatment options). Within ISBT a division can be made between Low-Dose Rate (LDR) and High-Dose Rate (HDR) treatments. The main difference between both types is that for LDR-ISBT lower dose rates (60 mGy / minute) are applied for a prolonged period (about 6–7 days) whereas HDR-ISBT applies a higher dose rate (1200 mGy / minute) for a much shorter period (10 times lasting about 5 minutes each). It has been estimated that the global market for brachytherapy has reached a value of about US$ 680 million in 2013, for which both LDR- and HDR-ISBT segments accounted for about 70% [4].

Although LDR-ISBT is commonly applied in the treatment of mobile tongue cancer, HDR-ISBT does have certain advantages. For instance, the treatment duration for each session is drastically reduced (whereas LDR-ISBT patients have to wear the radioactive implants during the whole treatment period). Another advantage of HDR-ISBT is that due to the fact that it employs fractionated irradiation cancer cells are damaged, however, normal cells are more preserved compared to treatments applying a continuous irradiation regime. Additionally, the amount of radiation exposed to the medical personnel for LDR-ISBT (e.g., due to initial insertion and periodical inspection of the implants) can be avoided. However, one concern with HDR-ISBT is that although it has the advantage of delivering a high dose locally to a specific target for a short time, the to-be-irradiated areas are usually in close proximity to the gingiva and the mandible (as tongue carcinomas typically arise at the lateral border of the tongue). These regions have been shown to be sensitive to the dose rate of radiation even up to the point that severe complications such as osteoradionecrosis or osteomyelitis may occur [5]. For instance, it has been shown that patients receiving a total dose of more than 50–60 Gy have a greater chance of developing mandibular bone complications [6]. Additionally, it has been reported that these complications are even more prevalent when EBRT (external beam radiotherapy) is applied as a combination treatment [7,8]. Another study indicated that the incidence rate for osteoradionecrosis is relatively high. About 8.2% of patients taken from a large sample (N = 830) documenting a 30-year period (i.e. between 1969 and 1999) displayed osteoradionecrosis to a variety of degrees [9]. Additionally, a recent study [10] indicated a mean weighted prevalence of 5.3% for osteoradionecrosis following brachytherapy based on a meta-analysis of several studies published between 1990–2008.

To prevent severe complications such as osteoradionecrosis, which has been reported to be extremely painful for patients [7,11], the dose to normal tissue needs to be attenuated as much as possible. This can be achieved by increasing the distance between the irradiation source and the mandible by means of a “spacer” (a prosthesis typically made of resin or silicon impression material placed between the mandible and the tongue). It has been shown that when the thickness of the spacer is increased to >10 mm the radiation dose can be reduced by about 65% or more for both radium and iridium treatment regimes using LDR-ISBT [7,12]. Additionally, Obinata et al. [13] in a clinical study using LDR-ISBT found that 18.2% of patients having a spacer less than 10 mm thick developed osteoradionecrosis whereas when the spacer was minimally 10 mm thick typically no osteoradionecrosis occurred. In all, it seems reasonable to assume that the thicker the spacer the more radiation attenuation will be obtained.

However, the space between the tongue and the mandible is quite limited (also depending on tumor size) and in several experimental and clinical studies using low dose-rate radioisotopes [14–16] it has been suggested that spacers using embedded metals known for their radiation shielding properties (i.e. having a high atomic number) should be beneficial to further reduce irradiation exposure without increasing the thickness to impractical proportions. However, to date the most prevalent spacer type in treating tongue cancer with interstitial brachytherapy remains to be the unshielded acrylic resin spacer (e.g. [17–19]) and to the best of our knowledge, there has been no report assessing the importance of modular spacers to be used in HDR-ISBT.

The aim of this paper is therefore to show how modular spacers with metal shielding can be readily constructed from suitable dental materials and to point out their clinical importance. Furthermore, we demonstrate how modular spacers can be used in a medical setting, starting with the treatment planning (using 3D-CT), how metal shielding can be applied after the planning stage, and how these spacers can be applied during the treatment stage for mobile tongue cancer using HDR-ISBT.

## Materials and Methods

**Fig 1:**
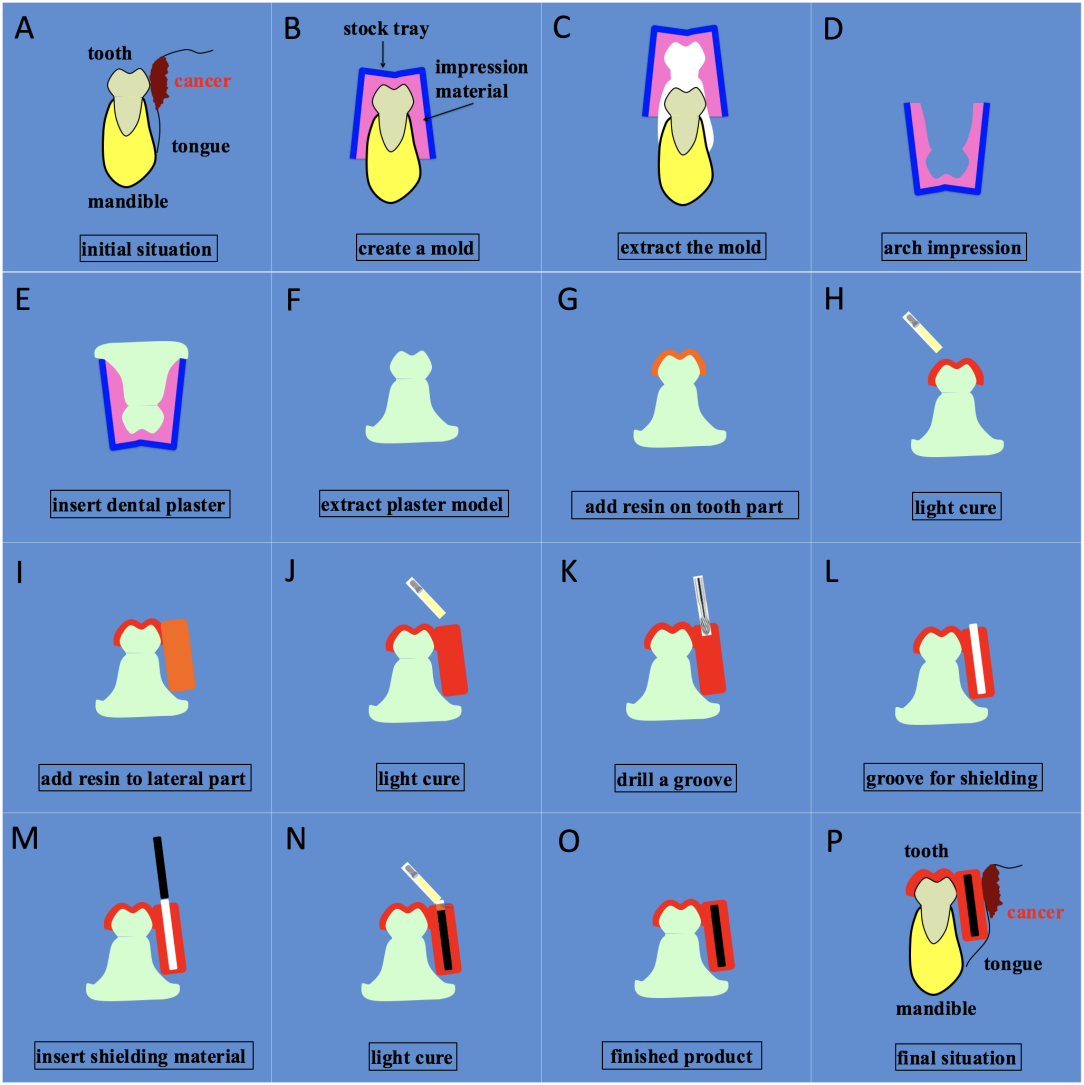
Construction steps of a modular spacer containing shielding material.

The construction of the modular spacer consists of several steps depicted in Fig 1. First, a teeth arch impression is taken using an alginate impression material (COE Alginate; GC America Inc., Chicago, IL, USA) commonly used in dentistry (B-C; 1 minute) and a plaster model is constructed from the arch impression (D-F; 1 hour).

**Fig 2:**
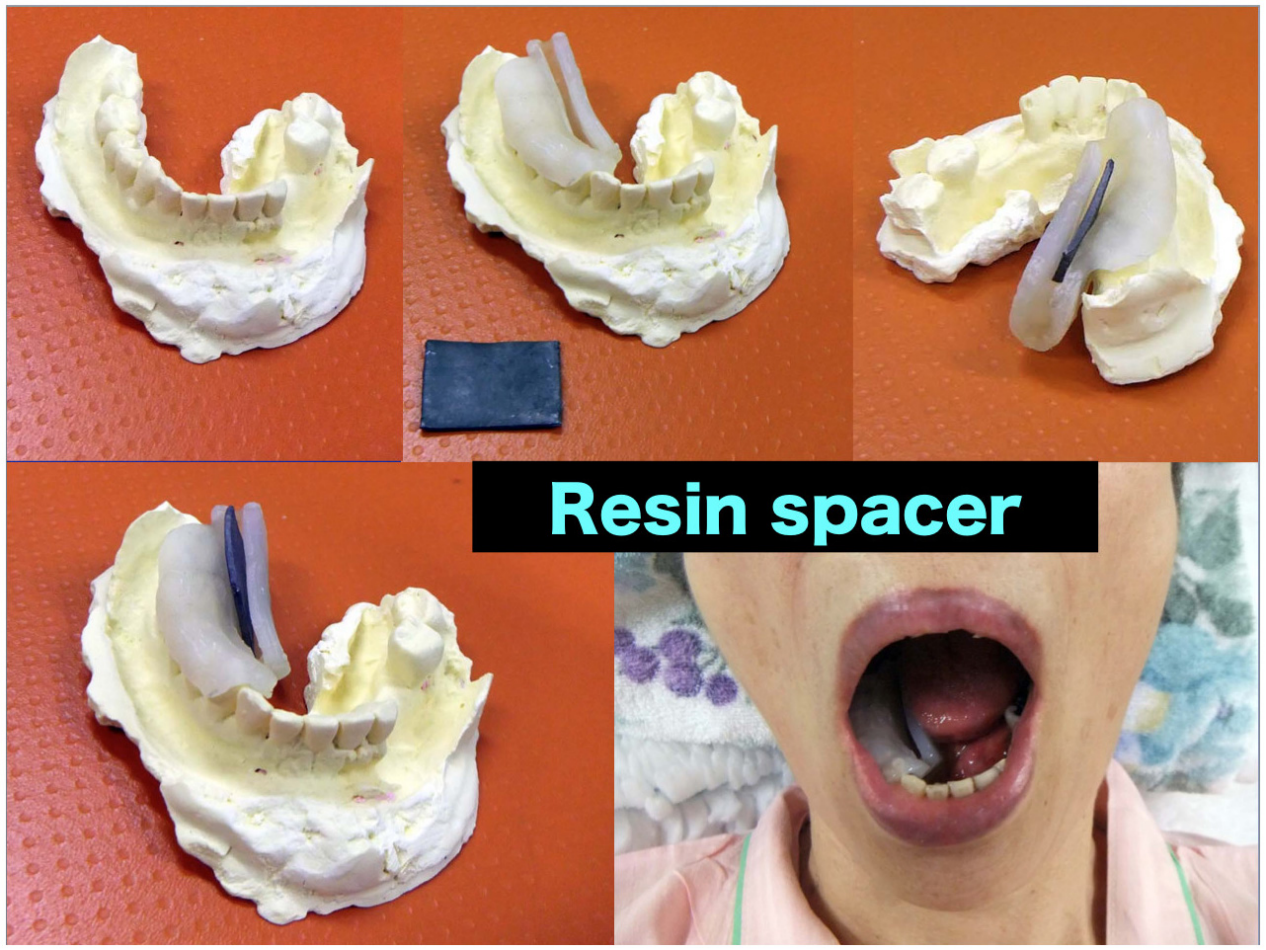
The modular spacer: inserting and holding the lead shield.

Subsequently, sheet-type light photo-polymerization cured resin (Splint-Resin LC; GC Corporation Ltd, Tokyo, Japan; effective atomic number of 12.5 with a density of 1.2 g/cm3) commonly used in dentistry is added to the plaster model (G; i.e. tooth part; 1 minute) and is hardened with a light cure (H; 5 minutes). The next step is to add resin to the lateral part of the spacer (1 minute) with a subsequent light cure (5 minutes), which will form the barrier between the tongue and the mandible (I-J). Next, a groove will be drilled in the lateral part (K-L; 5–10 minutes) that later will hold the shielding material (e.g. lead with an atomic number of 82 and a density of 9.8 g/cm3). At this point the spacer is ready to be used for 3D CT planning. Once the planning is finished the required amount of shielding material will be inserted and resin will be added to the top with subsequent light curing (M-N; 5 minutes) which will result in the final product (O). If the spacer requires additional adjustment, resin (and/or shielding) can be easily added or removed from the spacer during the actual planning or treatment session. In case the treating physician opts to order the modular spacer from a dental workshop (instead of producing it on-site) it is still possible and easy to perform the last steps (M-O; Fig 2) on-site instead of sending the spacer back to the workshop (which usually takes a long time) to seal the groove holding the shielding material (see Fig 2).

## Dose Rate Reduction Assessment for the Modular Spacer According to TG-43

To evaluate the dose rate reduction for a wide range of potential configurations we performed a number of computer simulations. We first arranged a HDR radiation source (Iridium-192) in either a single or double plane (for large tumors). Following Tsai et al. (6) and Kakimoto et al. [20] who reported that 50–60 Gy would be the maximum dose, we set 60 Gy at 5 mm offset from the lateral source. We simulated the absorbed dose at 10, 15, 20, 25, 30, 35, 40 mm distance from the lateral source combined with a varying lead thickness (0–5 mm).

Absorbed dose calculations are based on the mathematical formulas described in the protocol “Task Group No. 43 for Ir-192 sources” (TG-43) by the American Association of Physicists in Medicine [21,22]. The dwell weights of all sources were augmented by geometric optimization [23] to make the dose distribution homogeneous. The dwell times were calculated to achieve the reference point given a prescription dose of 60 Gy. In terms of the attenuated dose through lead, a half-value-layer of 3 mm was used for the lead material [24]. Other regions except for the lead were assumed to be in water when calculating the absorbed dose.

### Results

**Fig 3:**
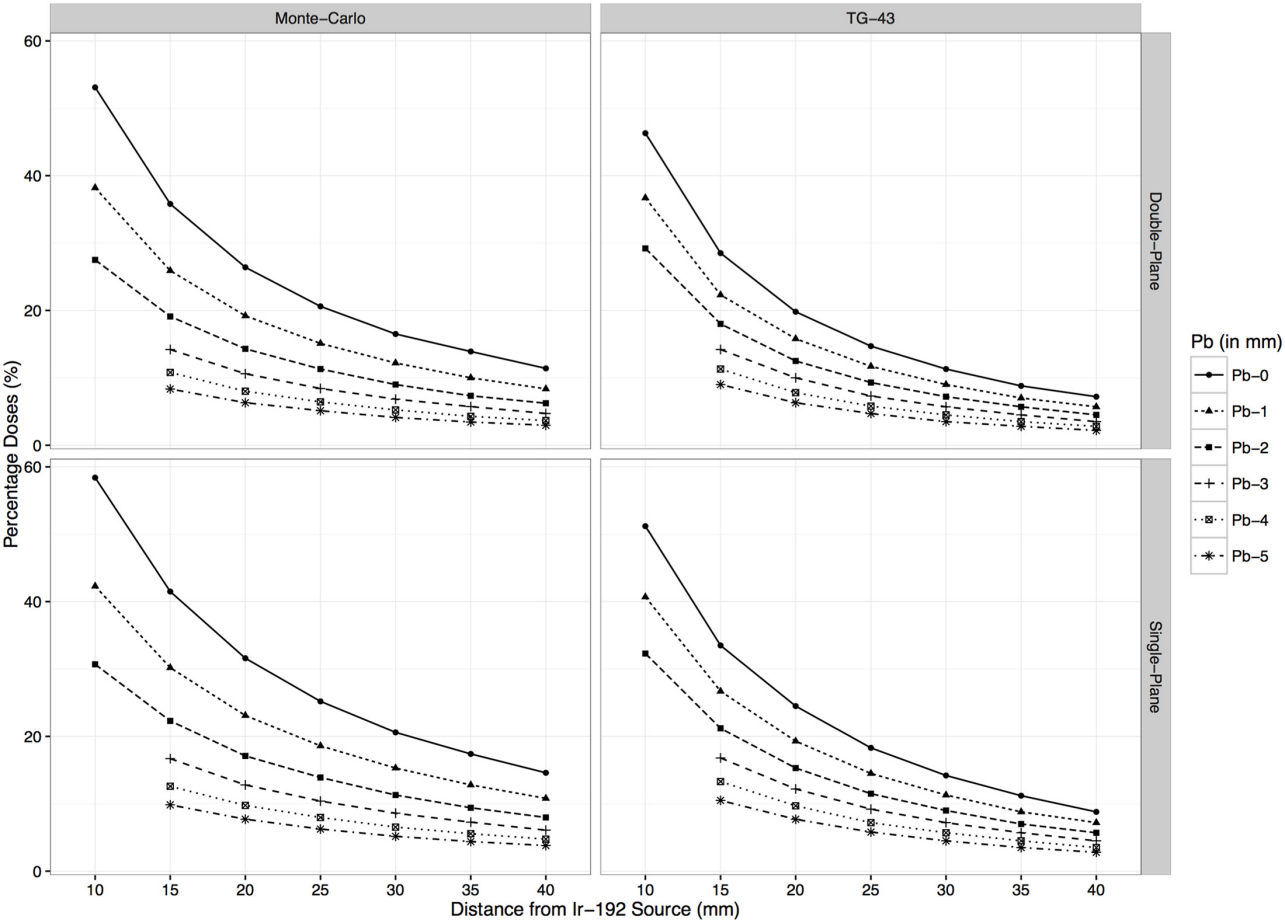
Percentage doses for various spacer types (Pb in mm) and distances (in mm) split out by plane and simulation type (Monte-Carlo vs. TG-43).

As expected we found that when lead was added significantly more shielding was obtained and absorbed doses dropped dramatically (see Fig 3; right pane). This effect was most pronounced on shorter distances to the source. For example, for the shortest distance between the mandible and the reference point (e.g. 10 mm from the source or 5 mm from the 60 Gy reference point) the dose reduction can go from 51.2% (30.7 Gy) of the 60 Gy dosage at the reference point for Pb-0 (indicating a 5 mm modular spacer with no lead shielding) to 40.7% (24.4 Gy) if 1 mm lead shielding would have been applied with 4 mm resin spacer (2 mm resin on both sides).

## Dose Rate Reduction Assessment Based on Monte-Carlo (MC) Simulations

To further validate the TG-43 results we also implemented a Monte-Carlo simulation [25] to estimate the simulated photon energy absorption at each measurement point using the same configurations. In our simulations, data pertaining to the specific photon interaction cross-section were derived from [26,27], the data of the relative probability and the energy of generating gamma-rays from Ir-192 were refereed to a radioisotope database [28] and the number of generated gamma-rays (histories) was ~1.0 x 10^8. The results of the MC simulations can be seen in Fig 3 (left pane) as well as in Table C in the S1 Appendix. A Welch two-sample t-test indicated that they were not statistically different from the earlier TG-43 simulations, t(152.3) = 1.29, p = 0.2.

## Discussion

When treating tongue carcinoma, high dose-rate interstitial brachytherapy (ISBT) is an important treatment option. Typically, ISBT administers a dose of a particular strength locally to a to-be-irradiated target. However, the mandible and gingiva due to their close proximity to the irradiation site may inadvertently also receive some part of this dose. This could lead to severe complications such as osteomyelitis and even osteoradionecrosis. This paper reports how to construct a modular spacer that can be used for the treatment of tongue carcinoma using HDR-ISBT.

We started out by specifying the basic steps necessary to construct the modular spacer and subsequently assessed its effectiveness in attenuating the dosage to the mandible. The results suggest that this type of spacer significantly reduces the dosage received by the mandible and allows for a large variety of configurations to be used in a clinical setting. Constructing a spacer in a modular way is not only quick (total construction time is less than 2 hours) but also allows physicians to rapidly adjust to a wide range of clinical conditions by manipulating the range of the thickness of the spacer itself, the thickness of the resin and the thickness of the lead shield. Take for instance an extreme situation in which there is almost no space between the mandible and the tongue due to excessive tumor size. In such extreme cases it is often opted to not implement a spacer. However, with a modular spacer it is technically possible to create a small 5 mm spacer with 2 mm resin on each side holding a 1 mm lead shield in its groove. Therefore, a small modular spacer with a 1mm lead shield would be strongly recommended, as it will greatly attenuate the absorbed radiation dose by the mandible compared to a spacer-less situation.

The effectiveness of adding metal shielding to clinical spacers was investigated in 1994 by Fujita et al. [14] who by using Lipowitz-metal (2 mm) embedded in a fixed spacer obtained marked dose reduction effects between metal lined- and unlined resin spacers in an experimental and clinical study that used low dose rate radioisotopes. Although for both radium and iridium irradiation sources there were significant dose reductions, the effect was most prevalent for iridium most likely due its weak gamma ray energy emissions [12,14].

One difference between Fujita et al. and the current study is that their spacer is likely intended to be used in LDR-ISBT in which exact 3D CT planning is not as central as in HDR-ISBT (as the radioisotope typically cannot be exactly set in the planned location). Additionally, their spacer involves the use of Lipowitz-metal (a.k.a. Wood’s metal) which has the advantage of a relatively low melting point (70C) making it easy to handle as a melting facility is not required. However, Lipowitz-metal also has certain significant disadvantages such as the fact that it contains cadmium which is extremely toxic (even in trace quantities). Another advantage to use lead is its higher atomic number which delivers higher protection against irradiation. Lead is also cheaper and softer than Lipowitz-metal and as most radiation treatment facilities have a lead-melting device we opted to use lead in combination with our modular spacer.

It is also important to point out that in Fujita et al. ([14], p.592) it can be seen that relative dose reduction rates (DRR) are not uniform for each patient (some patients’ DRR diverge by more than 15%). This indicates that some patients may benefit from additional shielding whereas standard shielding may be sufficient for others. These data were acquired using Thermoluminescent Dosimetry rods (TLD rods) to measure the absorbed dose and the measures reported in the current paper fit well with them. For instance, when comparing an unshielded resin spacer on 15 mm from the source (i.e. 10 mm from lateral reference point) the value dropped from 60 Gy to 20.1 Gy (~66.5% reduction) in our data. This closely matches the ~66% dose reduction rate reported in Fig 3 of Fujita et al. ([14]; p. 591) using actual TLD rods. Similarly, when adding 2 mm of lead shielding at this distance the absorbed dose dropped to 12.7 Gy (~79% dose reduction) in our simulation corresponding to the ~75% dose reduction rate of Fujita et al. [14] obtained (as they used another shielding material, i.e. Lipowitz metal under different conditions, the number is not exactly similar). See Tables A-C in the S1 Appendix (and Fig 3) for all the information containing our absorbed dose calculations, their reductions and the effects of lead and single or double plane sources (note that double-plane sources distribute the dose over a larger surface and have therefore overall lower values than single-plane sources).

Although this conclusion is clear, it is surprising that few papers dealing with ISBT and carcinoma of the tongue reported to have used lead-lined modular spacers. Most reports on LDR-ISBT involve a resin-only [17] or lead-only spacer (e.g. [29,30]) and this pattern is similar for HDR-ISBT (e.g. [31,32]).

We believe that by using a modular spacer there is much to be gained both in the planning and treatment stages. For instance, a 10 mm resin only spacer, although providing satisfactory protection to a certain extent [13], is clearly surpassed by a lead-lined modular spacer in terms of the absorbed dose by the mandible (see our estimations in the results section and Tables A-C in the S1 Appendix). Additionally, mandible lead shields may produce various side effects of having lead (a toxic compound) in the oral cavity even when it is applied for a limited amount of time. Additionally, the specific nature of the interface between tissue and the surface of lead is important due to potential secondary scatter radiation emitted from the surface of a lead-only spacer. For that reason, it is more favorable to create some distance as present in the current modular spacer (e.g. when enveloping the lead into the resin).

### Using the modular spacer in clinical practice

**Fig 4:**
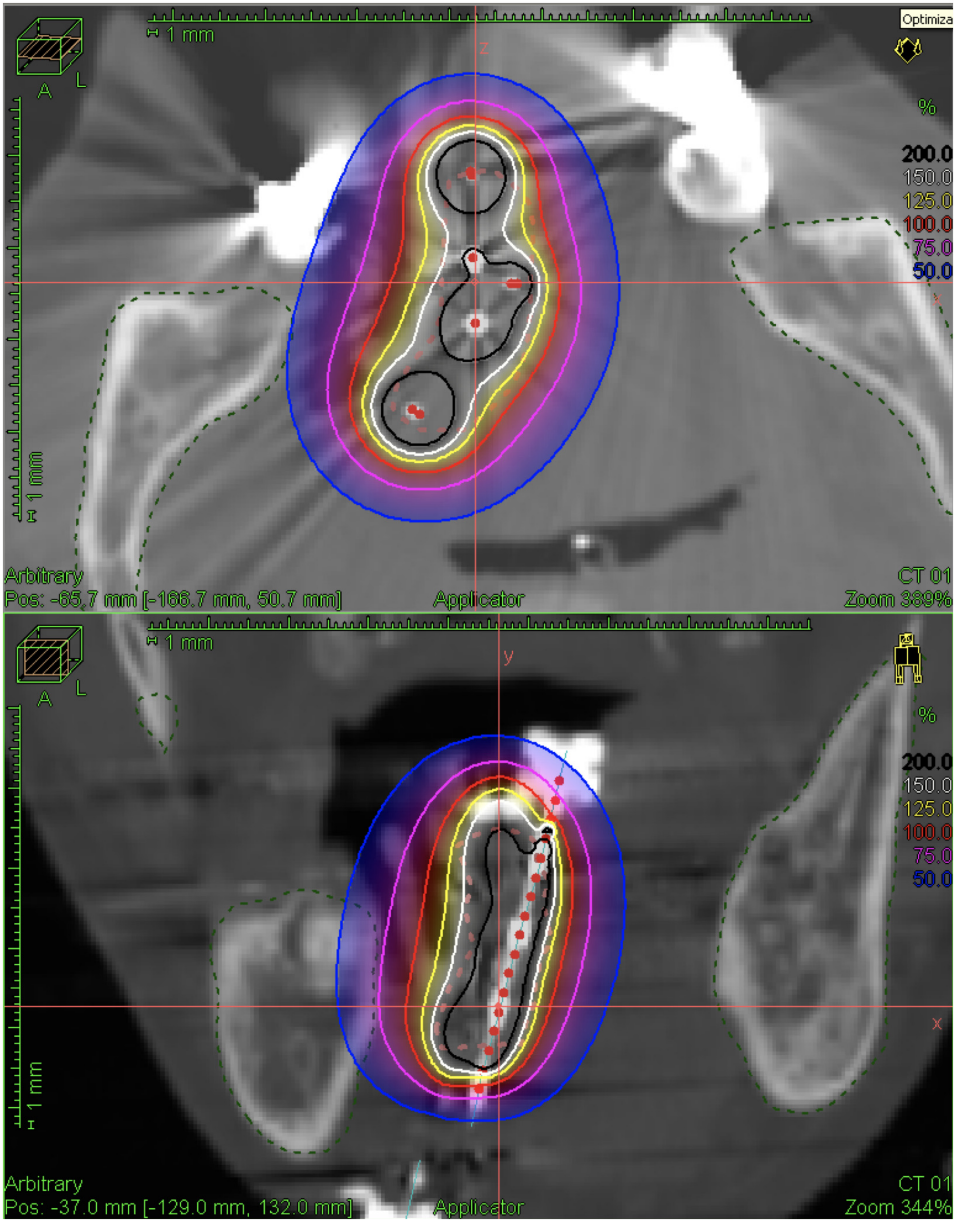
Modular spacer during 3D-CT planning without lead-shield (i.e. eliciting no artifacts).

Nowadays, treatment planning (especially in HDR-ISBT) relies heavily on 3D CT images, which have become indispensible to assess the individual adjustment and optimization of the dose distribution for each patient’s irradiation site [23,33,34]. One point of concern is that metals having high atomic numbers (such as lead or Lipowitz-metal) produce large metal artifacts on 3D CT images (i.e. due to scatter, beam hardening, and photon starvation; e.g. [35,36]). This poses difficulties for treatment planning if shielding devices such as custom designed mandibular lead shields [29] or non-modular (e.g. ready made) shielded spacers are involved in the planning and treatment. Ideally, the spacer should already be present when using 3D-CT planning, as its size and position should be taken into account to determine the exact dose distribution for the to-be irradiated sites (see Fig 4).

Therefore, using the currently described modular spacer one can first determine the appropriate size of the spacer, its resin thickness and shielding factor according to each specific patient’s circumstance. Then create the spacer (e.g. following the protocol provided in this paper), perform pre-treatment 3D CT planning (with the unshielded spacer present), and finally add the necessary amount of shielding material before treatment. In this way all the benefits from precise 3D-CT dosage-distributional planning are preserved whilst keeping sufficient protection of the mandible due to the modular shielding.

The modular spacer is well suited for both low-dose (LDR) and high-dose rate (HDR) ISBT. For instance, in LDR-ISBT patients receive a small fraction of the irradiation dose for a long time (e.g. continuously for over a week). In this situation patients have to wear the spacer constantly, which is quite uncomfortable and detrimental to oral hygiene. Additionally, medical personnel also are exposed to radiation and patients are isolated from their surroundings for a significant amount of time. In this situation typically the thinner the spacer the more comfortable the treatment is for the patient. By adding metal shielding to the modular spacer adequate dose attenuation can be obtained whilst keeping the spacer comfortable. Conversely, for HDR-ISBT the patient will receive a much higher dose for a shorter amount of time. In this case comfort is somewhat less important as the irradiation time is much shorter (e.g. 5 minutes per session). Therefore, a thicker spacer with increased shielding can be opted for to obtain more protection for a short period of time.

### Study limitation

We have started to implement the modular spacer in 14 patients in our hospital starting from two years ago. Although these patients were observed for a relatively short period of time, both late (~2 year) and acute complications (such as redness, erosion, and ulcers in the gingiva) have not been observed to date.

However, it will take time (5–10 years) to see the actual clinical outcomes of this spacer in terms of its shielding effects from osteoradionecrosis (as this condition usually takes years to develop) but the absorbed dose estimations are very encouraging and closely follow the TLD data from Fujita et al. [14].

## Conclusion

We believe that a modular spacer (allowing for on-site insertion or removal of metal shielding as well as further customization of the resin) represents a significant improvement over lead-only or resin-only non-modular spacers. Although the positive points of metal-lined spacers were already introduced roughly 20 years ago [13] they are still not in standard practice during the treatment of tongue carcinoma. The benefits nevertheless are very clear: First of all, the modular spacer is not overly complicated to construct and the construction process overall takes less than 2 hours. Secondly, it provides the opportunity for on-site modification without the strict necessity to return it to a dental workshop (which would take time). This becomes important for instance when patients’ circumstances unexpectedly change during the treatment. Lastly, it provides important options for the planning (3D-CT) and treatment processes (e.g. wide range of sizes), which are not feasible using resin-only or lead-only spacers. Lastly, modular lead-lined spacers provide excellent protection against particular harmful side effects of ISBT and therefore help to prevent serious complications such as osteomyelitis and osteoradionecrosis.

In conclusion, the modular spacer represents an important addition to the tools available for the planning and treatment of mobile tongue cancer using HDR ISBT and is readily applicable for usage on a wide scale in the treatment of tongue cancer.

## Supporting Information

S1 Appendix Table A. TG-43 simulations (in Gy) for single/double plane concerning five lead (Pb) thickness variations at various distances (in mm) from the lateral source. Table B. The effect of lead shielding (based on TG-43). Table C. Monte-Carlo simulations (in Gy) for single/double plane concerning five lead (Pb) thickness variations at various distances (in mm) from the lateral source.
(PDF)
