# Supplementary material for: Preventing Complications from High-Dose Rate Brachytherapy when Treating Mobile Tongue Cancer via the Application of a Modular Lead-Lined Spacer
Source: PLoS One. 2016 Apr 29;11(4):e0154226. doi: 10.1371/journal.pone.0154226 (PMC4851388; doi:10.1371/journal.pone.0154226)
Supplement: S1 Appendix — Table A. TG-43 simulations (in Gy) for single/double plane concerning five lead (Pb) thickness variations at various distances (in mm) from the lateral source. Table B. The effect of lead shielding (based on TG-43). Table C. Monte-Carlo simulations (in Gy) for single/double plane concerning five lead (Pb) thickness variations at various distances (in mm) from the lateral source. (PDF) [file pone.0154226.s001.pdf]

Table A – *TG-43 simulations (in Gy) for single/double plane concerning five lead (Pb) thickness variations at various distances (in mm) from the lateral source (the numbers in brackets represent the percentage doses to the 60 Gy reference dose at the lateral border of source at 5 mm).*

	**Single-Plane**							
	**Distance from Centre of Lateral Source**							
**Pb-Thickness**	**5**	**10**	**15**	**20**	**25**	**30**	**35**	**40**
**0**	600 (100)	307 (51.2)	201 (33.5)	147 (24.5)	110 (18.3)	85 (14.2)	67 (11.2)	53 (8.8)
**1**	600 (100)	244 (40.7)	160 (26.7)	116 (19.3)	87 (14.5)	68 (11.3)	53 (8.8)	43 (7.2)
**2**	600 (100)	194 (32.3)	127 (21.2)	92 (15.3)	69 (11.5)	54 (9)	42 (7)	34 (5.7)
**3**	600 (100)	-	101 (16.8)	73 (12.2)	55 (9.2)	43 (7.2)	34 (5.7)	27 (4.5)
**4**	600 (100)	-	80 (13.3)	58 (9.7)	43 (7.2)	34 (5.7)	27 (4.5)	21 (3.5)
**5**	600 (100)	-	63 (10.5)	46 (7.7)	35 (5.8)	27 (4.5)	21 (3.5)	17 (2.8)
	**Double-Plane**							
**0**	600 (100)	278 (46.3)	171 (28.5)	119 (19.8)	88 (14.7)	68 (11.3)	53 (8.8)	43 (7.2)
**1**	600 (100)	220 (36.7)	134 (22.3)	95 (15.8)	70 (11.7)	54 (9)	42 (7)	34 (5.7)
**2**	600 (100)	175 (29.2)	108 (18)	75 (12.5)	56 (9.3)	43 (7.2)	34 (5.7)	27 (4.5)
**3**	600 (100)	-	85 (14.2)	60 (10)	44 (7.3)	34 (5.7)	27 (4.5)	21 (3.5)
**4**	600 (100)	-	68 (11.3)	47 (7.8)	35 (5.8)	27 (4.5)	21 (3.5)	17 (2.8)
**5**	600 (100)	-	54 (9)	38 (6.3)	28 (4.7)	21 (3.5)	17 (2.8)	13 (2.2)

Table B – *The effect of lead shielding (based on TG-43). Values indicate the percentage doses (in %) compared to the Gy value at Pb-thickness = 0 (mm) for that distance.*

	**Single-Plane**							
	**Distance from Centre of Lateral Source**							
	**5**	**10**	**15**	**20**	**25**	**30**	**35**	**40**
**Pb-Thickness**								
**0**	600	307	201	147	110	85	67	53
**1**	600	79.5%	79.6%	78.9%	79.1%	80.0%	79.1%	81.1%
**2**	600	63.2%	63.2%	62.6%	62.7%	63.5%	62.7%	64.2%
**3**	600	-	50.2%	49.7%	50.0%	50.6%	50.7%	50.9%
**4**	600	-	39.8%	39.5%	39.1%	40.0%	40.3%	39.6%
**5**	600	-	31.3%	31.3%	31.8%	31.8%	31.3%	32.1%
	**Double-Plane**							
**0**	600	278	171	119	88	68	53	43
**1**	600	79.1%	78.4%	80.5%	79.5%	79.4%	79.2%	79.1%
**2**	600	62.9%	63.2%	63.6%	63.6%	63.2%	64.2%	62.8%
**3**	600	-	49.7%	50.8%	50.0%	50.0%	50.9%	48.8%
**4**	600	-	39.8%	39.8%	39.8%	39.7%	39.6%	39.5%
**5**	600	-	31.6%	32.2%	31.8%	30.9%	32.1%	30.2%

Table C – *Monte-Carlo Simulations (in Gy) for single/double plane concerning five lead (Pb) thickness variations at various distances (in mm) from the lateral source (the numbers in brackets represent the percentage doses to the 60 Gy reference dose at the lateral border of source at 5 mm).*

				**Single-Plane**				
	**Distance from Centre of Lateral Source**							
	**5**	**10**	**15**	**20**	**25**	**30**	**35**	**40**
**Pb-Thickness**								
**0**	600 (100)	350 (58.4)	249 (41.5)	190 (31.6)	151 (25.2)	124 (20.6)	104 (17.4)	88 (14.6)
**1**	600 (100)	254 (42.3)	181 (30.2)	139 (23.1)	112 (18.6)	92 (15.3)	77 (12.8)	65 (10.8)
**2**	600 (100)	184 (30.7)	134 (22.3)	103 (17.1)	83 (13.9)	68 (11.3)	56 (9.4)	48 (8.0)
**3**	600 (100)	-	100 (16.8)	77 (12.8)	62 (10.4)	52 (8.6)	44 (7.3)	37 (6.0)
**4**	600 (100)	-	76 (12.6)	59 (9.8)	48 (8.0)	39 (6.5)	33 (5.6)	29 (4.8)
**5**	600 (100)	-	59 (9.8)	46 (7.7)	38 (6.3)	31 (5.2)	26 (4.4)	23 (3.8)
				**Double-Plane**				
**0**	600 (100)	319 (53.1)	215 (35.8)	158 (26.4)	124 (20.6)	99 (16.5)	83 (13.9)	68 (11.4)
**1**	600 (100)	229 (38.2)	155 (25.9)	115 (19.2)	91 (15.1)	73 (12.2)	60 (10.0)	50 (8.4)
**2**	600 (100)	165 (27.5)	115 (19.1)	86 (14.3)	68 (11.3)	54 (9.0)	44 (7.3)	37 (6.2)
**3**	600 (100)	-	85 (14.2)	64 (10.6)	50 (8.4)	41 (6.8)	34 (5.7)	28 (4.7)
**4**	600 (100)	-	65 (10.8)	48 (8.0)	39 (6.4)	31 (5.2)	26 (4.3)	22 (3.7)
**5**	600 (100)	-	50 (8.3)	38 (6.3)	31 (5.1)	25 (4.1)	21 (3.4)	18 (3.0)
